# Towards an Integrative Account of Potential Mechanisms Mediating the Path From Sleep Dysfunction to Hallucinations

**DOI:** 10.1093/schbul/sbaf107

**Published:** 2025-10-06

**Authors:** Bryony Sheaves, Vanessa L Cropley, Peter Moseley, Peter W R Woodruff, Georgia Punton, Clemens Speth, Jana Speth, Peter Meerlo, Sanne G Brederoo

**Affiliations:** Department of Experimental Psychology, University of Oxford, Oxford, OX3 6NW, United Kingdom; Oxford Health NHS Foundation Trust, Warneford Hospital, Oxford, OX3 7JX, United Kingdom; Centre for Youth Mental Health, The University of Melbourne and Orygen, Parkville, VIC, 3052, Australia; Department of Psychology, University of Northumbria, Newcastle-Upon-Tyne, NE1 8ST, United Kingdom; Division of Population Health, School of Medicine and Population Health, University of Sheffield, 30 Regent Street, Sheffield, S1 4DA, United Kingdom; Department of Psychology, Durham University, Durham, DH1 3LE, United Kingdom; Institute for Medical Humanities, Durham University, Durham, DH1 3LE, United Kingdom; Institute of Psychology, University of Würzburg, 97070 Würzburg, Germany; Institute of Psychology, University of Würzburg, 97070 Würzburg, Germany; Neurobiology Expertise Group, Groningen Institute for Evolutionary Life Sciences, University of Groningen, Nijenborgh 7, 9747 AG Groningen, The Netherlands; University Center of Psychiatry, University Medical Center Groningen, University of Groningen, 9713 GZ, The Netherlands

**Keywords:** sleep, insomnia, hallucinations, voices, mediators, mechanisms

## Abstract

**Background:**

Sleep dysfunction shares a bidirectional relationship with hallucinatory experiences, with the strongest path from sleep dysfunction to the occurrence of hallucinatory experiences. This review aimed to identify potential mechanisms through which sleep dysfunction leads to hallucinations.

**Study Design:**

A narrative review was conducted across 4 levels of explanation: phenomenology (via lived-experience accounts), psychology, neural networks, and neurophysiology.

**Study Results:**

Relatively few studies have directly tested underlying mechanisms linking sleep dysfunction to hallucinations, particularly at the levels of neural networks and neurophysiology. There is good support for stress as a mediator between sleep dysfunction and hallucinations. Stress was a plausible mechanism across levels of explanation and was supported by sleep manipulation studies in non-clinical populations. Inflammation of the nervous system is affected by sleep loss, which in turn impacts the brain connectivity underpinning hallucinatory experiences. Lived-experience accounts identified 3 novel mechanisms, all of which are meaningful to people with lived experience of hallucinations: source monitoring, mental resilience, and reasoning skills. Quantitative studies show these mechanisms are impacted by sleep loss, but the full causal path from sleep dysfunction to hallucinations via these mechanisms requires testing.

**Conclusions:**

Key priorities for future research are to (1) test stress as a mediator in clinical populations experiencing hallucinations, with stress assessed across the levels of explanation simultaneously; (2) carry out experimental tests of novel potential mediators identified in this review (eg, source monitoring, inflammation, prefrontal cortical networks); and (3) identify potential moderators that might explain individual differences in the lived-experience accounts of the effect of sleep dysfunction on hallucinations.

## Introduction

“[*After a bad night of sleep the voices*] *would be a lot louder, and I would hear them more*”

(Voice-hearer “V13”,^1^)

Over the past decade, manipulation studies have provided sound support for the causal role of sleep disruption in the occurrence of hallucinatory experiences. Disrupting sleep in healthy volunteers induces sub-clinical increases in hallucinatory experiences.^2^ Conversely, treating sleep disruption in students with insomnia lessens hallucinatory experiences, with over a third of the reduction in hallucinations attributable to improvements in insomnia.^3^ Further support for the causal argument comes from the temporality of effects: sleep disruption typically precedes, and is predictive of, a first episode of psychosis.^4^ While the relationship between insomnia and hallucinatory experiences is bidirectional,^1^^,^^5-7^ longitudinal evidence shows that the strongest path is from insomnia to later hallucinatory experiences.^5^ The current review seeks to take the causal argument further by identifying potential mediating mechanisms that might account for the path from sleep dysfunction to hallucinations.^8^

We use a levels-of-explanations approach to identify potential causal pathways between sleep dysfunction and hallucinations from a phenomenological, psychological, neuronal, and neurophysiological perspective. This results in a candidate set of potential causal mechanisms, which in some cases may act across domains to explain the path from sleep dysfunction to hallucinations. Rather than definitively answering the question of why sleep dysfunction leads to hallucinations, the current study is meant as a hypothesis-generating account, guiding future research in advancing our understanding. “Sleep dysfunction” serves as an umbrella term covering the breadth of deviations from healthy sleep (eg, sleep deprivation/restriction, nightmares, and clinical insomnia). The most common sleep disorders, experienced by around half of people with psychosis, are insomnia and problematic nightmares.^9-11^ The majority of existing mechanistic research has focused on insomnia and sleep loss specifically; hence, this is the key focus of the review. The review sought to answer the following 5 research questions.

1. How do people with lived-experience of hallucinations describe the path from sleep dysfunction to hallucinations?
2. Which psychological factors mediate the association between sleep dysfunction and hallucinations?
3. How does neuronal connectivity affected by sleep make the brain more susceptible to hallucinations?
4. How do neurophysiological changes resulting from sleep dysfunction increase the risk of experiencing hallucinations?
5. Are there common pathways that hold across the four levels of explanation?

## The Phenomenological Level

Lived-experience accounts identify how sleep and hallucinations are linked from a first-person perspective, and importantly, which mechanisms are meaningful and plausible to those experiencing hallucinations. Lived-experience accounts of voice-hearing (auditory verbal hallucinations) and their relation to sleep are drawn from three qualitative interview studies.^1^^,^^6^^,^^7^

The first potential mechanistic path is that sleep disruption induces negative affect, which in turn increases negative voice content: “*that’s down to me sleeping I suppose… like, my mood. It’s just [if my mood isn’t so good], just more violent […] as in, the things [the voices] say*” (V10^1^). For some voice-hearers intense negative affect results from nightmares, which in turn triggers voices: “*it’s very difficult once they’ve [the nightmares] started and you are awake and then the voices start interfering with you*.” (N2, qualitative interview participant^12^). Improved sleep following psychological sleep treatment can lead to positive changes in the voice-hearing experience: “*Calming my voices in my mind down as well because I am sleeping a lot more*” (participant 5^7^). This mechanistic path chimes with quantitative findings that fragmented sleep leads to longer and more negatively perceived utterances of hallucinated voices.^13^

The second mechanistic route described in lived-experience accounts is that sleep dysfunction leads to decreased mental resilience, leading to lower perceived control over voices and subsequently an increase in their frequency: “*when I haven’t slept […] I struggle to like even make them stop. I don’t have the energy, so […] it’s like it’s all bombarding me at once […]*” (V10^1^). Feeling worn down due to sleep dysfunction alters the response to voices: “*When I’m tired it gets worse because I don’t have the strength to fight them as much*” (participant 1^7^). In quantitative tests, being worn down—a construct comprising mental defeat, low confidence in relation to voices, and fatigue—has been associated with more listening to and believing derogatory and threatening voice content.^14^

The third potential mediating mechanism between sleep and hallucinations is cognitive processing, such as source monitoring. Voice-hearers describe sleep as being a prerequisite for their ability to use logic in assessing whether or not a voice is “real”: “*how tired I am affects how logically I can think about whether it’s my voices or not […] so I think sleep has got a lot to play with it um. Whereas when I’m in quite a good routine with my sleep and stuff I can use the rationale, and the logic and stuff to work out whether it’s real or not. Whereas when I’m tired I don’t have that capability*.” (V5^1^)*.* Some participants described frequent decisions about whether they could rely on what their senses were telling them (ie, “*whether it’s real or not*”). The ability to monitor the source of sensory information and the executive functions that underpin decision making are plausible cognitive mediators of the relationship between sleep disruption and believing negative voice content.^14^

While voice-hearing is the most commonly investigated hallucinatory experience in qualitative accounts, there are also briefer examples of visual hallucinations being impacted by sleep disruption: *“Like to see something yesterday [a visual hallucination], was I think that was down to tiredness […], I was exhausted. Which is again why the sleep thing is so important.*” (V5^1^). To date, mediating mechanisms between sleep and visual hallucinations (or indeed any other sensory domain) have not been identified from qualitative research, but are a worthwhile area for future research.

It is important to note that the qualitative accounts support individual differences in the effect that sleep disruption has on hallucinations. For some voice-hearers, sleep disruption increased voice frequency: “*definitely that’s one of my main triggers, not getting enough sleep*” (V6^1^). However, others are clear that there is no noticeable link between sleep and voices: “*There’s never really been a pattern to be honest*” (V9^1^). Equally, for some voice-hearers, sleep is a time for respite and escape from voices,^6^^,^^7^ but this is not the case for voice-hearers who regularly experience nightmares, within which voices can occur.

In summary, negative affect, low mental resilience (feeling “worn down”), and source monitoring arise from lived-experience accounts as potential mechanistic pathways from sleep dysfunction to hallucinations. The majority of lived-experience accounts investigating the role of sleep in hallucinations pertain to voice-hearing (as opposed to hallucinations in other modalities), and the diversity of the accounts highlight the existence of individual differences.

## The Psychological Level

In line with lived-experience accounts, negative affect is recognized as a key mediator of the sleep-psychosis relationship.^15^ Stress (rather than symptoms of depression and anxiety more generally) might be particularly important in mediating the relationship. In Reeve et al.’s^2^ sleep restriction experiment with healthy volunteers, levels of hallucinatory experiences (assessed across multiple sensory domains) were significantly higher after restricted (4 hours for 3 nights) vs standard sleep. Self-reported stress mediated 43.4% of the relationship between sleep loss and hallucinatory experiences. Symptoms of depression and anxiety worsened following sleep loss, but were not significant mediators in this non-clinical group. Similarly, data from a general population group showed that while insomnia at time one significantly increased the odds of new experiences of voice-hearing 18 months later, the size of the direct longitudinal relationship remained largely the same when depression and anxiety were controlled for.^16^ However, initial longitudinal evidence in a clinical group suggests that depressive and anxiety symptoms should not be discounted as relevant mediating mechanisms. In a group diagnosed with non-affective psychosis, depressive symptoms mediated 80.5% of the relationship between insomnia and later hallucinations in all sensory modalities, and anxiety mediated 72.1% of the relationship.^5^ An interventionist causal model^17^ approach could determine whether treating sleep improves hallucinations via improving depression and anxiety at clinical levels of severity.

Lived experience accounts highlight the importance of cognitive processes in deciding whether to rely on what a voice is saying. This is described as an intentional process and involves holding in mind multiple pieces of relevant information and inhibiting irrelevant information in order to reach a decision about the veracity and source of the perceptual experience (a voice vs external source). The process is likely supported by working memory, inhibitory control, and source monitoring processes. Intentional cognitive inhibition refers to the ability to consciously and deliberately suppress non-relevant material from working memory.^18^ A lab-based sleep deprivation study found that false alarms on an intentional inhibition task increased following 24-hours of wakefulness, and decreased after a recovery sleep.^19^ Separately, a number of studies have shown that people reporting hallucinatory experiences make more false alarm responses on an inhibitory control task (ie, fail to suppress irrelevant stimuli) than non-hallucinating participants.^20-22^ Testing the full mechanistic path from sleep disruption to hallucinations via inhibitory control is a clear area for further research. The role of working memory in the sleep-hallucinations relationship has been experimentally tested in general population groups, confirming that sleep loss impairs working memory;^2^^,^^19^ however, there was no support for a mediating role of working memory in the effect of sleep loss on the occurrence of hallucinatory experiences.^2^ The relationship from sleep disruption to believing negative voice content, via working memory, has not been assessed.

There is evidence supporting the theory that voice-hearing results from inner speech being misattributed to an external source, that is, a problem of source monitoring.^23-25^ Meta-analyses indicate that clinical and non-clinical participants who hallucinate are more likely to incorrectly recall a spoken word as heard^26^ although this result has not been replicated in general population groups.^20^^,^^21^^,^^27^ Sleep deprivation affects source memory ability,^28^ and napping after encoding memories reduces source monitoring errors.^29^ Future, studies could usefully ascertain whether these findings reflect the well-established effects of sleep dysfunction on memory consolidation more broadly^30^ or source memory ability specifically. Also needed are studies that manipulate sleep and assess whether source monitoring ability mediates the relationship with hallucinations.

There is evidence that people prone to hallucinations experience a perceptual bias which increases the influence of prior (top-down) information over bottom-up sensory input (“over-weighted priors”).^26^^,^^31^^,^^32^ This bias is thought to underpin the occurrence of hallucinations, and could theoretically be affected by sleep. Two studies with non-clinical participants aimed to directly assess this, differing in the assessment used for perceptual processing. Both studies found that these attentionally driven top-down processes may be resilient against the effects of sleep deprivation.^2^^,^^19^ This suggests that changes in perceptual processing may not account for the relationship between sleep and hallucinatory experiences—at least in non-clinical groups—but this is an area that clearly warrants further research.

In summary, the strongest support has been found for a mediating role of stress in the pathway from sleep dysfunction to hallucinatory experiences across sensory domains. Data is inconsistent regarding depression and anxiety as candidate mediators. It is possible that they are relevant mediators at clinical levels of severity, but this requires testing in causal-interventionist designs. Deterioration in cognitive inhibition and source monitoring abilities result from sleep loss, but there are no tests of whether this results in more hallucinatory experiences, or results in voices being more believable. Non-clinical data indicate that source monitoring (reliant on conscious attentional processes) is negatively affected by sleep loss, but the full causal chain from sleep loss to hallucinatory experiences via source monitoring requires testing.

## The Neural Networks Level

Altered functional activity related to neural network connectivity is a recognized feature of sleep dysfunction as well as hallucinations.^33-35^ This aberrant connectivity has been identified within and across distributed networks involving perception, memory, and emotion. However, to our knowledge, there are no studies that directly assess the role of neural connectivity mechanisms in mediating the link between sleep dysfunction and hallucinations. Therefore, our hypotheses on mechanistic pathways underlying the relationship between sleep and hallucinations at the level of functional connectivity are derived from studies that report similar neural network characteristics of hallucinations on the one hand, and of sleep dysfunction on the other hand.

A large body of neuroimaging research shows that hallucinations are underpinned by intrinsic brain signals in sensory cortices,^36-38^ also subserving the sense that those perceptions arise from external space.^39^ In addition, evidence supports the hypothesis that hallucinations arise from release of those sensory signals by reduced top-down inhibition from higher-order centers such as frontal areas and their connections with the thalamus.^40-43^ Whereas normally such higher-order areas exert top-down inhibition on intrinsic sensory cortical activity, diminished connectivity between higher-order centers and sensory cortices may lead to hallucinations. Different lines of evidence support the notion that sleep dysfunction may give rise to hallucinations by affecting (1) sensory networks, (2) emotion-related areas such as the amygdalae, and (3) lack of higher-order inhibition of those areas by frontal regions and the thalamus.

Regarding sensory networks, Zhao et al.^44^ reported significantly increased intrinsic connectivity following sleep-deprivation in the resting sensorimotor network. A number of neuroimaging studies show evidence for both increased sensory activity and decreased connectivity of higher-order areas following sleep dysfunction, again consistent with the model of reduced top-down control of the sensory processes that drive the hallucinatory experience. Specifically, functional neuroimaging studies following sleep deprivation report reduced resting-state connectivity of cortico–cortical and subcortical–cortical networks,^34^^,^^45^ coupled with increased connectivity within sensorimotor and limbic networks.^44^^,^^46^ Further support comes from studying groups with insomnia: a review by Fasiello et al.^47^ reported increased functional connectivity within sensory/sensorimotor (including auditory and visual pathways), limbic, salience, and default mode networks, whereas decreased connectivity was most prominent in frontal networks.

Regarding the amygdala and frontal areas, functional connectivity between the medial-prefrontal cortex and regions responsive to negative emotion (ie, amygdala) was shown to be affected by sleep deprivation.^48^ Sleep loss essentially disrupts the usual inhibitory function of the medial-prefrontal cortex on the amygdala during the processing of emotionally salient information. Indeed, sleep deprivation most strongly affects the functional connectivity in prefrontal regions.^49^ It may be that the sleep deprived prefrontal cortex is less able to assert its influence upon “lower level” cortical systems that have behavioral as well as emotional consequences. For instance, poor sleep quality was related to greater affect-related impulsivity among adolescents, but only in those with low functional connectivity between the prefrontal cortex and default mode network.^50^ Ben Simon and colleagues report that the interaction between sleep deprivation and the magnitude of anxiety increase is accounted for by changes in medial-prefrontal cortex activity and associated medial-prefrontal cortex connectivity.^51^ Hence, the loss of functional connectivity of the prefrontal cortex following sleep disruption may be the neurological underpinning of negative affect that was shown to mediate the relationship between sleep loss and hallucinations (as reported at the psychological level).^2^

Further support for a link between the prefrontal cortex and amygdala comes from emotional memory paradigms. Total sleep deprivation results in a deterioration in the recollection of positive and neutral stimuli, yet recollection of negative stimuli is preserved.^52^ Sleep deprivation is associated with the recruitment of an amygdala-cortical network when recollecting negative, but not positive stimuli. Hence, the lack of prefrontal inhibition of sensory cortical activity and amygdala in humans reported by Yoo et al.^48^ may mediate a bias towards negative emotional valence in sleep deprived individuals and could potentially contribute to the distressing emotional tone of hallucinations during states of sleep dysfunction. These observations may also help understand the strong association between sleep disturbance and depression. For instance, a study by Cheng et al.^53^ reported that functionally connected networks mediate the association between depressive symptoms and poor sleep quality. They specifically highlighted the functional connectivity between the lateral orbitofrontal cortex, cingulate cortex, precuneus, angular gyrus, and temporal cortex as important in mediating the connection between sleep and depressed mood. These regions comprise a complex network linking higher cortical control regions to sensory systems^54^ that includes the cingulate cortex suggested to modulate spontaneous auditory activity in acoustic silence as demonstrated using functional magnetic resonance imaging (MRI).^38^

The impact of sleep dysfunction on frontal networks may also affect hallucinations via changes in cognition. Lived-experience accounts highlighted the role of sleep disruption in impairing the ability to decide on the source (an inner voice vs external source) and, therefore, veracity of voices. Sleep deprivation results in deficits in the top-down allocation of attentional resources,^55^ which results in impairment in orienting to a location, where a target is expected to appear,^56^^,^^57^ the capacity for sustained attention,^58^ and for selective attention.^57^ Following sleep deprivation, reductions in activity in the dorsolateral pre-frontal cortex and intraparietal sulcus are observed, which contribute to these attentional performance deficits.^55^ To our knowledge, the effect of reduced neural activity in regions which subserve attention on hallucinatory experiences has not been investigated. It is plausible that disrupted top-down allocation of attention mediates the path between sleep loss and greater listening to negative voices.^14^

In addition to frontal areas, thalamic functioning is a likely candidate for altered inhibition on intrinsic activity in sensory areas and the amygdala, and is strongly implicated in sleep. Shao et al.^45^ showed reduced thalamocortical functional connectivity after prolonged (36 hours) wakefulness. Furthermore, disorders associated with hallucinations, such as schizophrenia, are associated with a reduction in sleep spindles,^59^^,^^60^ which are generated within the thalamus. Sleep spindles are distinct but transient brain oscillations (9-16 Hz) during sleep, that result from dynamic interactions between thalamic reticular nucleus and thalamocortical relay neurons, which then generate the typical oscillatory spindling activity within thalamo-cortical pathways.^61^ Sleep spindles are thought to play an important role in gating sensory processing, stabilizing sleep, and supporting memory consolidation and cognitive function.^62^^,^^63^ Notably, in patients with schizophrenia, spindle deficits have also been shown to correlate with positive symptoms including hallucinations.^64^ While a reduction in spindles during the sleep phase may not be directly responsible for the occurrence of hallucinations during the subsequent waking phase, a spindle deficiency may reflect an underlying impairment in thalamic function and a disrupted thalamocortical connectivity. This impaired connectivity in turn might disturb sensory gating and processing of external and internal signals that could then contribute to the emergence of hallucinations.^59^

In summary, altered neural connectivity is a hallmark of both sleep dysfunction and hallucinations. Sleep dysfunction disrupts connectivity in higher-order areas like the frontal cortex and thalamus, which normally inhibit intrinsic sensory activity. This disruption can lead to hallucinations by increasing connectivity in sensory networks and reducing top-down control. Furthermore, emotional regulation is affected by sleep dysfunction, again disrupting the inhibitory function of the medial-prefrontal cortex on the amygdala. This might contribute to the negative emotional tone of hallucinations (reported in lived-experience accounts), and account for a drop in mood following sleep loss. Sleep spindles, generated in the thalamus, are crucial for sensory processing and cognitive functions. In schizophrenia, spindle deficits correlate with hallucinations, indicating impaired thalamic function and connectivity, and further supporting the role of dysconnectivity of higher-order control centers in hallucinations, following dysfunctional sleep.

## The Neurophysiological Level

While data on specific neurophysiological mechanisms underlying sleep dysfunction and hallucinations is limited, one could build a case based on (1) available knowledge regarding disorders that are associated with hallucinations, such as psychosis and schizophrenia and (2) whether the mechanisms proposed for such disorders are sensitive to restricted or disrupted sleep. Such mechanisms at the level of neurophysiology include (re)activity of the neuroendocrine stress and immune systems, inflammation, neuronal dysconnectivity and neurochemical perturbations.

Altered regulation and activity of the neuroendocrine stress response is a key vulnerability factor for the development, maintenance, and exacerbation of psychotic illness.^65^ Meta-analytic evidence points to elevated circulating levels of cortisol in people with first-episode psychosis,^66^^,^^67^ and blunted cortisol reactivity,^68^ together thought to reflect a chronic or “tonic” hypothalamic-pituitary-adrenocortical (HPA) hyperactivation and associated loss of physiological capacity to respond to acute stressors.^68^ Yet the relevance of neuroendocrine dysregulation to clinical symptoms, including hallucinations, remains unclear, with mixed relationships between cortisol and positive symptoms reported.^65^

Numerous studies have shown that sleep dysfunction is also often associated with mild increases in the activity of the major neuroendocrine stress systems, that is, the autonomic sympatho-adrenal system and the HPA axis.^69^^,^^70^ Specifically, sleep deprivation or sleep disruption is associated with increases in autonomic sympathetic activity as reflected in an increased level of catecholamines, heart rate, and blood pressure,^71^^,^^72^ as well as altered HPA axis regulation^69^ with higher basal levels of cortisol and increased cortisol responses to stress.^70^^,^^73^ Sensitization of the HPA axis might be a critical mechanism linking inadequate sleep to stress-related pathology, including impairments to mental health.^69^^,^^70^

Importantly, sleep dysfunction not only affects the regulation of neuroendocrine stress systems directly but also influences the perception of stressors at a cognitive level, whereby poorer sleep is associated with lower resilience to stress.^69^^,^^74^ It is thus plausible that stress dysregulation at both the neuroendocrine level and psychological level, and their complex interplay, may contribute to the relation between sleep dysfunction and hallucinations. Specifically, sleep dysfunction might decrease the psychological resilience and increase the physiological responses to daily stressors, which may increase the risk of developing clinically relevant psychotic symptoms. In support of this, clinical voice-hearers show dysregulated physiological stress-function compared to non-clinical voice-hearers,^75^ but experience similar levels of stressors, such as victimization,^75^ and individuals with prodromal or attenuated psychosis are more likely to show HPA hyperresponsiveness to daily stressors.^76^^,^^77^

On the level of the immune system, prospective longitudinal studies and meta-analyses show that chronic sleep dysfunction is associated with, and predicts, higher daytime levels of known indicators of inflammation, such as C-reactive protein (CRP) and interleukin 6 (IL-6).^78^ Initial findings demonstrate that these inflammatory markers can be reduced when sleep problems are adequately treated.^79^ In patients diagnosed with schizophrenia, poorer sleep is cross-sectionally associated with inflammatory markers.^80^ Alterations in CRP and IL-6 are associated with changes in psychotic symptoms over time.^81^ And in a recent cohort study using data from the Avon Longitudinal Study of Parents and Children (ALSPAC), higher levels of IL-6 in late childhood were shown to partially mediate the association between persistent shorter sleep duration across early childhood and subsequent psychotic disorder or psychotic experiences in young adulthood.^82^ Although this study cannot imply causality, it provides preliminary support for a mechanistic path from sleep dysfunction to psychotic experiences via inflammation. Intriguingly, while sleep dysfunction may promote inflammatory mechanisms (eg, via microglia) in the central nervous system directly,^83^ there is emerging evidence that peripheral inflammation can perturb brain connectivity, particularly within default mode and salience networks,^84^^,^^85^ contributing to downstream effects on behavior.^81^ In this context, it is noteworthy that cross-sectional relationships have been reported between peripheral IL-6 levels and default mode network connectivity in a pooled sample of schizophrenia and controls,^86^^,^^87^ where IL-6 mediated the relationship between early-life stress and brain connectivity.^87^

Studies discussed in the previous section suggest that, at the neural network level, aberrant connectivity of subcortical and cortical networks is a shared feature of hallucinations and sleep dysfunction. At the neurophysiological level, such changes in connectivity might be related to sleep dysfunction-induced changes in the number, size, shape, and strength of synapses. Experimental studies using animal models have shown that sleep dysfunction (including deprivation and fragmentation) is associated with changes in hippocampal morphology and neurogenesis.^71^^,^^88-90^ Although data are limited, microglial activation reported following sleep disruption in animal models may result in increased phagocytosis, or elimination, of synapses,^91^ which if persistent, could contribute to over-pruning of neural networks. Interestingly, exaggerated pruning of synapses^92^ and dysconnectivity of neural networks,^93^ are longstanding neurobiological models of schizophrenia, and convergent evidence points to synaptic pathways being integral to conferring schizophrenia risk.^94^ Specifically, positron emission tomography studies have shown increased synaptic density in sensory cortices and decreased synaptic density in superior frontal areas in people at risk for psychotic symptoms.^95^^,^^96^ These findings are in broad agreement with the notion that disorders associated with hallucinations are characterized by differentially altered functional connectivity in sensory and higher-order brain regions, and supports the view that this might be in part related to, or exacerbated by, dysfunctional sleep.

Given that aberrant dopamine signaling remains a leading theory of the pathogenesis of psychosis,^97^ it would be remiss not to consider dopamine as a possible mechanism linking sleep dysfunction and hallucinations. The dopamine hypothesis characterized by excessive dopamine activity in the thalamocorticostriatal network^97^ has been mechanistically linked with hallucinations by causing a perceptual bias towards prior (top-down) expectation.^98^ Sleep deprivation (or extended wakefulness) is also associated with alterations in dopamine signaling in humans and animals as characterized by a downregulation in D2/D3 receptors,^99^^,^^100^ increased expression of D1 receptors^101^ and dopaminergic transmission^101^^,^^102^ in midbrain and striatal regions. Adaptations in dopamine signaling may further modulate the neurobehavioral and neurophysiological consequences of sleep loss.^103^ As such, the role of dopamine in the link between sleep dysfunction and hallucinations is likely to be complex. Aberrant dopamine signaling may contribute to both sleep disruption (or extended wakefulness) and hallucinations (ie, via a shared mechanism underpinning both insomnia and hallucinations). In addition, given the bidirectional relationship between dopamine and sleep systems, whereby dysregulation of these systems exacerbates each other,^104^ aberrant dopamine signaling might represent a mediating mechanism between sleep dysfunction and hallucinations.

It is important to keep in mind that direct evidence for the putative neurophysiological mechanisms linking sleep dysfunction and hallucinations is lacking. The above neurophysiological mechanisms are also likely highly interactive at multiple explanatory levels; for example, dopamine can regulate inflammatory responses and vice versa,^105^ and neuroendocrine stress systems and immune regulation may impact neuronal connectivity and brain function.^106^^,^^107^ Together, interactions between these mechanisms might contribute to neurophysiological pathways linking sleep dysfunction and hallucinations.

## Discussion

The aim of this review was to generate hypotheses regarding mediators of the path from sleep dysfunction to hallucinations, across different levels of explanation. In reviewing the literature on the sleep-hallucination relation, it has become clear that relatively few studies have been devoted to direct testing of underlying mechanisms linking sleep to hallucinations, particularly with regards to neural networks and neurophysiology. Despite this, by evaluating known mechanisms associated with both sleep dysfunction and hallucinations, we were able to identify a number of potential mediating mechanisms of the sleep-hallucination relation across the four levels of explanation. Returning to the research questions, the following potential mediating mechanisms could be identified from our review.

**Figure 1:**
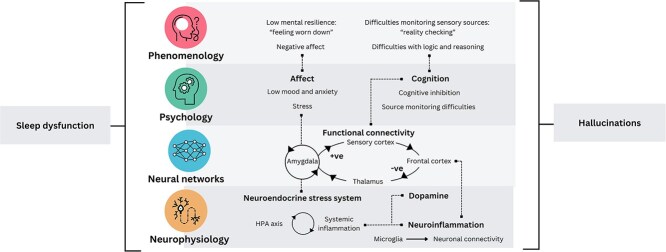
Potential mechanistic pathways from sleep dysfunction to hallucinatory experiences.

1. On the phenomenological level, several potential mechanisms connecting sleep dysfunction to hallucinations arose from lived-experience accounts. Negative affect was associated with more negative voice content. Low mental resilience (feeling worn down) was associated with higher voice frequency and less control over voices, and problems with source monitoring and reasoning were associated with difficulties assessing the veracity of voice-hearing experiences.
2. On the psychological level, strong support can be found for perceived stress as a mediating mechanism, and partial support was found for depression and anxiety. Inhibitory control and decreased source monitoring abilities are potential mechanisms underlying the sleep-hallucination relation which require further investigation.
3. On the neural network level, the link between sleep dysfunction and hallucinations could be mediated by dysconnectivity of prefrontal cortical control of intrinsic sensory and amygdala cortical activity.
4. On the neurophysiological level, sensitization of the HPA axis, inflammation of the nervous system, and altered dopamine signaling are neurophysiological changes resulting from sleep dysfunction that can be argued to increase the risk of hallucinations.
5. Several common pathways can be identified that hold across the 4 levels (Figure 1), which will be described in the following.

Support was found across the four levels for a mediating role of stress in the path from sleep dysfunction to hallucinations. Sleep dysfunction results in dysregulation of the neuroendocrine stress systems, which in turn is a key vulnerability factor for the development, maintenance and exacerbation of psychotic symptoms. This aligns with the finding that self-reported stress mediates the relation between sleep loss and the occurrence of hallucinatory experiences in a non-clinical group. Neuroimaging studies demonstrate that prefrontal inhibition of the amygdala is lacking in sleep-deprived people. This could provide the neural mechanism linking sleep dysfunction to negative affect. Neuroendocrine stress system dysregulation is associated with increased perceived stress, but in turn also lowers mental resilience to stressors. As persistent hallucinations (eg, threatening voices) can be an ongoing stressor for voice-hearers, neuroendocrine dysregulation following sleep dysfunction may lower resilience against experiences of distressing voices. Indeed, voice-hearers describe feeling less guarded against distressing voices when their sleep has been compromised. Causal-interventionist tests are now needed that assess whether stress holds as a mediating mechanism in a clinical population.

Another potential mediating mechanism encountered across the neural networks and neurophysiology levels of explanation is inflammation of the nervous system. An increase in inflammatory markers has been shown following sleep dysfunction. Such markers are also found to be heightened in people prone to hallucinating (ie, people with a schizophrenia-spectrum disorder). Furthermore, inflammation of the peripheral nervous system affects brain connectivity, potentially via over-pruning of synapses in neural networks related to frontal inhibition over sensory areas, as well as the default mode and salience networks.

Cognitive mechanisms (difficulties using logical thinking and “reality checking” voices) were identified as consequences of sleep problems in lived-experience accounts; however, very few studies have tested the full mechanistic path from sleep loss to hallucinations via cognitive mechanisms. Poorer source monitoring, working memory and intentional inhibition were consequences of sleep loss, but working memory was not a mediator between sleep loss and the occurrence of hallucinatory experiences. The full path from sleep disruption to hallucinations via source monitoring and intentional inhibition has not yet been tested. Initial tests indicate that psychological assessment of “top-down processing” was not affected by sleep loss. At the neural network level, sleep dysfunction caused aberrant connectivity of frontal inhibition over sensory brain areas. This is consistent with some lived-experience reports of lower perceived control over voices, and a subsequent increase in voice frequency.

Large increases in low mood and anxiety are observed following sleep loss,^2^ are clearly reported in lived-experience accounts and may be underpinned by reduced prefrontal-amygdala neural connectivity. Depression and anxiety mediated the majority of the path from insomnia to hallucinations in a longitudinal clinical group.^5^ However, symptoms of depression and anxiety were not significant mediators in a non-clinical experimental study.^2^ It is possible that symptoms of depression and anxiety are more prominent mechanisms in groups with clinical levels of hallucinations, but this hypothesis requires testing.

It is likely that some mechanisms act across different levels of explanation in parallel, for example, stress. There is also the possibility of reciprocal interactions between different mechanisms within levels. For example, within the neurophysiological level, there are complex bidirectional interactions between the neuroendocrine stress response and inflammation of the nervous system.^108^^,^^109^

## Future Studies

There is clear support for stress as a mechanism mediating the path from sleep disruption to the occurrence of hallucinatory experiences. Testing this mechanism across levels of explanation simultaneously would assess whether or not the psychological, neural network and neurophysiological stress mechanisms work in parallel. It is notable, however, that stress mediated just less than half (43.4%) of the path from sleep disruption to hallucinations,^2^ suggesting that there are likely other additional mediating mechanisms. Manipulation studies that assess the full causal path from sleep disruption to hallucinations are required to test the following hypothesized mechanisms: (1) source monitoring and intentional inhibition, (2) low mental resilience, and (3) inflammatory processes, which affect neuronal connectivity between prefrontal and sensory brain regions (ie, decreasing prefrontal inhibition of intrinsic sensory cortical activity). Additionally, lived-experience accounts highlight that sleep disruption potentially causes important changes in hallucinations beyond their occurrence alone. However, these have not been tested. Hypotheses include: (1) sleep disruption increases the degree of negative voice content, mediated by changes in depressive symptoms; and (2) sleep disruption increases the believability of voices, mediated by both feeling worn down (low mental resilience) and reasoning skills (underpinned by executive functioning).

Future studies should aim to ameliorate three notable limitations of the reviewed studies. First, hallucination measurement requires greater precision: studies on the neurophysiological level examine patient groups with schizophrenia-spectrum disorders rather than hallucinations specifically. At the psychological level, hallucinations were often assessed across sensory domains. It is as of yet unclear whether sleep dysfunction causes hallucinations across all sensory domains and equally unclear whether mediating mechanisms, such as stress, work in a supra-modal manner. Second, the measurement properties of some cognitive assessments, such as source monitoring, have low internal reliability.^107^ Third, sleep is assessed in a variety of ways, including insomnia severity, sleep dysfunction, sleep health, and sleep loss. While insomnia has been the key focus of the review, there are other common causes of sleep dysfunction for people experiencing hallucinations, such as problematic nightmares and excessive sleepiness.^9^^,^^10^^,^^110^ Lastly, it is possible that constructs across different levels of explanation do not truly align, for example: (1) lived-experience reports of difficulty tracking the source of perceptual experiences vs performance on source monitoring tasks; (2) attentionally driven top-down processes assessed at the psychological level (eg, the Ebbinghaus illusion task) and a lack of top-down inhibition and release of intrinsic sensory cortical activity at the neural networks level; and (3) neurophysiological vs psychological measures of stress. Lived-experience involvement in the design of experimental studies could increase the ecological validity of selected measures.

Lived-experience accounts demonstrate individual differences with regard to the relationship between sleep and hallucinations. For some voice-hearers, poor sleep inevitably leads to worsening of voice-hearing, while other voice-hearers observe that sleep has little or no effect on their hallucinations. This highlights the need to identify moderators which explain for whom, and under what circumstances sleep dysfunction leads to hallucinations. Identification of such moderators could explain some of the variable results found in this review. For example, it is possible that depressive symptoms are only a mediator of the path from sleep disruption to hallucinations in clinical populations, and on the neurophysiological level, gene polymorphisms, for example of dopamine signaling, could be responsible for differential outcomes of sleep dysfunction.^103^

The review was concerned with the key mechanisms underlying the sleep and hallucinations relationship. There are additional areas that warrant further research, for example: hallucinations are prevalent in groups experiencing narcolepsy (though narcolepsy is not common in groups with psychosis). The role of hypocretin deficits as a potential mechanism underpinning hallucinations occurring for this sub-group of people is worthy of further investigation. Additionally, it would be useful to understand the occurrence of hallucinations at sleep on/offset (hypnopompic/hypnogogic hallucinations). These may be more common for people with psychosis.^9^ It is possible that this is a by-product of long and fragmented sleep windows (ie, a greater number of transitions between sleep and wake), but this requires further research.

## Conclusion

For decades, clinical observations have noted that significant sleep deprivation leads to hallucinatory experiences.^111^ Manipulation studies in non-clinical populations have since confirmed that sleep dysfunction is indeed a causal factor in the occurrence of hallucinatory experiences.^2^^,^^3^ Sleep dysfunction is treatable in clinical populations experiencing hallucinations.^112^^,^^113^ Ascertaining the impact of sleep treatments on hallucinations in patient groups, and the route through which the treatment may act, could inform decisions about the degree of priority to give sleep treatment and the outcomes patients can expect from treatment. This review identified potential routes through which sleep dysfunction may alter hallucinatory experiences. There is good support for the role of stress in mediating the relationship between sleep dysfunction and hallucinations. The mediating effect of stress is plausible across multiple levels of explanation, further validating the finding. Inflammation of the nervous system is also a likely mechanism, which in turn impacts brain connectivity that underpins hallucinations. Comparison of lived-experience accounts with existing quantitative studies provided an opportunity to identify novel mechanisms that remain meaningful to people with lived experience of hallucinations. Source monitoring, mental resilience and reasoning skills are clear candidates arising from this that require further rigorous testing.
